# Assessing cumulative neighborhood effects on adult health

**DOI:** 10.1371/journal.pone.0213204

**Published:** 2019-04-24

**Authors:** Jason Fletcher, Daniel Jung

**Affiliations:** 1 La Follette School of Public Affairs, Department of Sociology, Institute for Research on Poverty, Center for Demography of Health and Aging, University of Wisconsin-Madison, Madison, Wisconsin, United States of America; 2 Department of Population Health Sciences, University of Wisconsin-Madison, Madison, Wisconsin, United States of America; University of Botswana, BOTSWANA

## Abstract

A straightforward technique to explore the “total effects” of neighborhoods on health outcomes is to compare the degree of similarity of outcomes of neighbors with those of non-neighbors. Several issues arise in interpreting these estimates around spatial and temporal definitions of “neighbors” and life course mobility patterns. Indeed, much work uses “cross- sectional neighbors,” which makes the interpretation of the estimates unclear because they combine short-term effects (for movers) and long-term effects (for stayers). This paper contributes to the literature by assessing the importance of measuring neighbor mobility as well as neighborhood selection. Using the Panel Study of Income Dynamics, we examine the extent to which having longitudinal measures of “neighbors” shapes estimates of neighborhood effects, and also use a negative test of neighborhood effects to assess the importance of neighborhood selection. Specifically, we estimate similarity in self-rated health of adults over 30 years old who live in the same county over various periods of time and find that “cross-sectional” neighbor definitions may understate neighborhood effect estimates by as much as 35%. However, when we contrast these health estimates with contemporaneous neighborhood “effects” on completed education, we find that much of the “understated” effects on health are likely related to selection effects rather than causal effects of neighborhoods.

## Introduction

Large literatures across many social science disciplines have attempted to examine the impacts of place on health processes. When pursuing this question, several issues of measurement and interpretation become apparent. Are we interested in short-term or long-term effects of place? Are we interested in overall impacts of place or specific aspects of place (e.g., poverty, health care access)? How might we separate the effects of place from the selection of place? The answers to these questions are consequential for interpreting the estimates in the literature.

For the subset of the literature that has explored “overall” impacts of place rather than effects of specific neighborhood measures, one framework that has been advanced is to assess the degree to which “neighbors’” outcomes are similar compared with those of non-neighbors. The idea is an extension of approaches in genetics that compare identical twins (who share 100% of their DNA) with fraternal twins (who share 50% on average) and reason that the extent to which identical twins have more similar outcomes than fraternal twins can be attributed to genetic factors. Related research has examined the “total effects” of families (combining both genetics and environments) by examining the degree to which siblings have similar outcomes. Solon, Page, and Duncan [1] then further extended these ideas to estimate the impacts of place during childhood on adult outcomes by examining the extent to which childhood “neighbors” had similar educational outcomes using the Panel Study of Income Dynamics (PSID). The level of similarity is typically interpreted as an “upper-bound” effect of place because it combines selection effects and causal effects. That is, because neighborhoods and neighbors are not randomly assigned, a portion of similarity is likely due to selection effects. Solon et al. [1] attempt to reduce the portion of the selection effects by adding control variables in their model that predict educational attainment and also predict place (e.g., parental income). Upper-bound estimates are useful in cases where the estimates are small because they can potentially rule out that place is a major determinant of the outcome. Upper-bound estimates that suggest a large effect of place are less useful because they are not able to separate causal and selection effects.

Subsequent work has used Add Health data to examine additional measures of adolescent development and alternative measures of neighbors [2] and expanded the analysis to adult outcomes [3]. One issue with these extensions is the use of “cross-sectional neighbors” in the Add Health data, which is used because the data do not measure place in a comprehensive way over time. Add Health currently has four waves of data over a period of 15 years That is, the extent to which neighbors have similar outcomes will depend on how long the pairs have been neighbors and the importance of short- versus long-term effects of shared exposures. A key concern about using “cross-sectional neighbors” is that the upper-bound estimates could be too small in cases where the neighbors have not lived in the neighborhood for very long. Thus, research that uses cross-sectional neighbors and finds small overall place estimates do not allow larger place effects to be ruled out, defeating a key advantage of the approach.

This paper contributes to the analysis of place using the approach of comparing neighbors in two ways. By using PSID data that have more complete longitudinal measures of place, we contrast estimates of the upper-bound effects on health for individuals who have lived in the same place for 1 to 10 years. Our results of this analysis suggest that using cross-sectional neighbors could underestimate the upper-bound effects of place on health by up to 35%. However, an issue with using long-term neighbors is that selection effects are likely to be more important since people who have remained in the same place for longer periods of time are a select group who might have worse health. These issues point to our second contribution.

We deploy a “negative test” to explore causal versus selection effects of place. Focusing on respondents over 30 years old, who have nearly universally completed their schooling, we estimate the importance of place for neighbors who have lived in the same place for 1 to 10 years on years of completed schooling. Since place during adulthood cannot affect completed schooling for adults, any increase in the estimated effect of place that occurs as we condition on the number of years remaining in a neighborhood must be due to selection. Indeed, we find that shifting from “cross-sectional neighbors” to “longitudinal neighbors” increases the estimates of place effects by 30% for an outcome that is predetermined, showcasing an important counteracting bias in using this research design.

## Methods

We use the Panel Study of Income Dynamics (PSID), a longitudinal household panel survey of a nationally representative sample of U.S. families. PSID measures economic, social, and health factors over the life course of families over multiple generations. These data were collected by the University of Michigan’s Institute for Social Research annually through 1997 and biennially thereafter since 1998 [4].

PSID respondents provide information about themselves, their spouse/partner, and all other family members living together who are considered as a family. In 1968, the PSID used a cluster sample design to develop a national sample of approximately 4,800 U.S. households [4]. As members of the sample families grow up, move out, and form their own economically independent households, they are interviewed separately, increasing the overall number of interviews conducted each wave. This trajectory led PSID to include data from nearly 9,000 families in 2015.

To define “neighborhood,” we use the restricted version of PSID, which provides household members’ census block and higher-level geographical identifiers in each wave. State and county Federal Information Processing Standards (FIPS) codes are used to define the neighborhoods where respondents live based on the county at the time of interview. Combining the 2-digit state FIPS code and 5-digit county FIPS code provides a unique state and county identifier. Respondents within the same county were considered to have lived in the same neighborhood for 1 year, and by matching with previous data, we identify respondents who have lived in the same neighborhood for longer periods of time. There is an inherent trade-off in most datasets between leveraging granular measures of neighborhood, such as census block or tract or school catchment areas, which are more accurate assessments of shared environments on one hand, and the small sample sizes of households in many datasets per neighborhood on the other hand. The PSID is not a large enough sample to use more granular measures than counties. We recognize that this level of aggregation likely reduces our ability to capture a portion of true neighborhood effects, however the focus of our analysis is to make comparisons across model specifications in how *neighbors* are temporally measured rather than in capturing granular neighborhood effects. This focus needs to be recognized in interpreting our key results below. As our primarily aim is to examine the upper-bound effects of neighborhood on health based on individuals who have lived in the same place, we restrict our analysis to counties with at least two residents.

We use self-reported general health to define health according to the survey question, “Would you say your health in general is excellent, very good, good, fair, or poor?” which was asked only of household heads and spouses of heads. Self-reported general health is known to be not only a strong predictor of adverse health events including mortality [5], but also basic quality of life measures [6]. As the study analysis could be affected by individuals who live in the same household, we restrict the sample to one person per household. As self-reported health was asked only of household heads and spouses of heads, we randomly select one person per household among households with both head and spouse. Also, as the same people were surveyed over years, we use the most recent response.

Other respondents’ characteristics including age, gender, race, and education level are included in the study. We consider age as a linear variable and categorize gender as male/female; race as white/black; and education level as not completed high school/12 years or completed high school/more than high school.

Considering the context of neighborhood could change across time, we utilize 2011, 2013, and 2015 data for health response. In addition, as young people are more likely to move their residence due to school and jobs, we focus on respondents who were age 30 years or older at the time of the survey. Finally, because we adjust for observable respondents’ characteristics including age, gender, race, and education level, we exclude the respondents that did not report those characteristics.

The empirical analysis is estimating the upper bound on the portion of the variance in health outcomes that can be attributed to the neighborhood, adjusted by age, gender, race, survey year, and education level. We utilize a random-effect multinomial logistic regression model where *P*(*R*_*ij*_ ≤ *k*) represents the probability of responding at or below the *k*^th^ level of the outcome variable; *β*_0*j*_ is the random intercept; *δ*_*k*_ represents the difference between the *k*th category and the preceding one; *j* designates neighborhood; *i* designates individual; *k* designates health outcome [*k* = 0 (poor), 1 (fair), 2 (good), 3 (very good), 4 (excellent)].

$$\eta_{kij}=\text{log}\left(\frac{P(R_{ij}\leq k)}{1-P(R_{ij}\leq k)}\right)=\beta_{0j}+\beta_{1j}X_{ij}+\delta_{k}$$ (1)

$$\beta_{0j}=\gamma_{00}+u_{0j}$$ (2)

The random intercept is consisting of *γ*_00_, the average log odds of reporting a given level or worse health status, and *u*_0*j*_, the random error term with *u*_0*j*_~*N*(0, *τ*_00_), where *τ*_00_ is the neighborhood level error variance.

We estimate the intraclass correlation (ICC) to estimate the upper-bound effect of neighborhoods on health. ICC is the ratio of the between-cluster variance to the total variance in the outcome that is not explained by other individual covariates. It informs us how much of the overall variation in the response is explained simply by clustering [7].

$$\frac{\tau^{2}}{\tau^{2}+\sigma^{2}}$$ (3)

Generally, the calculation for ICC follows the Eq (3), where *τ*^2^ denotes the between-cluster variance and σ^2^ denotes within-cluster variance (the variance of the residuals). However, as there is no direct estimation of the residuals σ^2^ on the first level in logistic regression, we follow the latent response formulation that is most widely used [8][9]. We can use the variance of logistic distribution, *π*^2^/3, as the level 1 variance, which allows us to have both the between-cluster variance and within-cluster variance on the same scale. According to the formula, we can calculate the ICC:

$$\frac{\tau^{2}}{\tau^{2}+\frac{\pi^{2}}{3}}$$ (4)

We estimate the longitudinal upper-bound effect of neighborhood on health by comparing the ICC estimates across individuals who have lived in the same place for more than 2, 4, 6, 8, and 10 years. Furthermore, to identify the effect of neighborhood selection, we run a negative test that examines the neighborhood effect on education level.

## Results

We summarize the background characteristics of 12,437 respondents in Table 1. In the sample restricted to age 30 years or older, the average period of living in the same neighborhood was nearly 13 years, and more than 8 out of 10 respondents reported their health as good or above. The analytic sample size is 8,262 after randomly selecting one person per household and including counties with at least two respondents. After applying the exclusion criteria, nearly 80% of respondents reported their health as good or above and the mean age was 50.4 years old. While the percentage of male, white, and highly educated respondents was reduced, respondents who were the head of the household increased.

**Table 1:** Panel Study of Income Dynamics. Descriptive statistics for sample: Overall sample and one per household.

Variable	30 or Older	One per Household	Two or More per County
*N*	12,437	8,666	8,262
Years of neighborhood (mean (sd))	13.0 (10.1)	13.3 (10.2)	13.6 (10.3)
Self-reported health (%)			
Excellent	14.9	14.5	14.5
Very Good	34.9	33.0	33.0
Good	31.7	32.1	32.0
Fair	13.5	14.7	14.9
Poor	5.1	5.8	5.7
Age (mean (sd))	50.3 (14.8)	50.4 (15.3)	50.4 (15.3)
Gender: Male (%)	43.8	42.2	41.9
Race: White (%)	67.0	63.0	61.9
Head relation: Head (%)	68.1	75.2	75.6
Survey year (%)			
2011	5.1	7.2	7.2
2013	10.4	11.1	11.1
2015	84.5	81.7	81.7
Education level (%)			
Lower than HS	12.9	14.6	14.7
Graduate HS	31.1	31.7	31.7
Higher than HS	56.0	53.7	53.6

Table 2 examines the upper-bound effect of neighborhood on self-reported health adjusting for age, gender, and race. Johnson, Schoeni, and Rogowski [10] use a similar approach and the same data but focus on comparing neighbors during young adulthood on older adult health outcomes [10]. The authors find correlations in self-reported health among neighbors during young adulthood of 0.24–0.33 (compared with spousal correlations of 0.46).

**Table 2:** The effects of neighborhood on health: Estimates of the percentage of the variation in adult self-rated health status attributable to county of residence.

	30 or older	32 or older	34 or older	36 or older
*N*	8,626	7,752	7,244	6,676
Overall	0.031* (-0.002–0.063)	0.029* (-0.005–0.062)	0.033** (0.002–0.063)	0.035* (-0.004–0.075)
*N*	7,005	6,649	6,305	5,889
County > 2	0.035** (0.004–0.067)	0.032** (0.003–0.061)	0.038** (0.004–0.071)	0.039** (0.001–0.078)
*N*	6,108	5,857	5,598	5,292
County > 4	0.035** (0.002–0.069)	0.034* (-0.001–0.069)	0.039** (0.004–0.075)	0.042** (0.000–0.084)
*N*	5,298	5,089	4,894	4,658
County > 6	0.038** (0.001–0.075)	0.037** (0.005–0.069)	0.041** (0.004–0.079)	0.044** (0.001–0.087)
*N*	4,675	4,480	4,311	4,127
County > 8	0.039** (0.003–0.075)	0.039** (0.002–0.075)	0.043** (0.005–0.082)	0.041** (0.003–0.080)
*N*	4,136	3,967	3,813	3,648
County > 10	0.046** (0.009–0.082)	0.046** (0.007–0.085)	0.050** (0.012–0.088)	0.048** (0.003–0.092)

Note:

*p<0.1,

**<0.05,

***<0.01

Controls: Age, Gender, Race, Survey year. *Columns* restrict the ages of the sample; *Rows* restrict the sample to respondents who have lived in the same county for a given number of years.

In the overall sample, the intraclass correlation is 0.03, which means 3% of the variance in self-reported health is attributed to the neighborhood (county). The estimates of the intraclass correlation continuously increase by nearly 50% as the time spent in the same neighborhood increases to more than 10 years. Generally, the neighborhood effect on health increases with older age groups.

Table 3 shows results of our main analysis that build on Table 2 by adding a control for education level. This is done to begin to examine the possibility of selection effects into neighborhood (based on educational attainment). Generally, comparing the results to the prior model (Table 2), the intraclass correlation in health decreases nearly 35% after adding an adjustment for education level. The results show that 2% of the variance in adult self-rated health status is attributable to neighborhoods (counties) when taking into account the controls in the analysis. Similar to prior analysis (Table 2), the intraclass correlation among those who lived in the same place for more than 10 years is more than 50% higher when compared to the overall sample. These estimates suggest that cross-sectional study design could underestimate the upper-bound effects of neighborhood on health by approximately 35% (but as much as 50%). A key result from this table is the lack of importance of selection effects, as measured by the change in coefficients down the rows of results after conditioning on education.

**Table 3:** The effects of neighborhood on health: Estimates of the percentage of the variation in adult self-rated health status attributable to county of residence, controlling for educational attainment.

	30 or older	32 or older	34 or older	36 or older
*N*	8,626	7,752	7,244	6,676
Overall	0.019 (-0.014–0.053)	0.017 (-0.016–0.050)	0.021 (-0.010–0.051)	0.022 (-0.009–0.054)
*N*	7,005	6,649	6,305	5,889
County > 2	0.022 (-0.009–0.054)	0.020 (-0.009–0.048)	0.024 (-0.009–0.057)	0.025 (-0.011–0.061)
*N*	6,108	5,857	5,598	5,292
County > 4	0.023 (-0.010–0.056)	0.021 (-0.015–0.057)	0.025 (-0.010–0.060)	0.026 (-0.006–0.058)
*N*	5,298	5,089	4,894	4,658
County > 6	0.025 (-0.010–0.061)	0.023 (-0.011–0.058)	0.027 (-0.011–0.065)	0.027 (-0.007–0.060)
*N*	4,675	4,480	4,311	4,127
County > 8	0.026 (-0.011–0.062)	0.026 (-0.014–0.066)	0.029 (-0.010–0.067)	0.027 (-0.009–0.063)
*N*	4,136	3,967	3,813	3,648
County > 10	0.031 (-0.007–0.068)	0.031 (-0.012–0.074)	0.034 (-0.005–0.072)	0.032 (-0.009–0.073)

Note:

Controls: Age, gender, race, survey year, education level. *Columns* restrict the ages of the sample; *Rows* restrict the sample to respondents who have lived in the same county for a given number of years.

While Table 3 did not appear to produce results suggesting that selection effects were a primary concern in the results from Table 2, we also pursue this issue in a second direction. Table 4 reports results from the *negative test* to explore the selection effects of neighborhood by focusing attention on an outcome (educational attainment) that can have no causal effect on adult neighborhood (recall the respondents are over age 30). The analysis results show that 9% of variance in education level was explained by neighborhood, and longitudinal neighbors increases the estimates of place effect by over 20%. However, considering the analysis focuses on people of age 30 and older who have likely completed their education, the increase in the estimated effect of place on completed education according to longitudinal neighbors should be interpreted as the effect of neighborhood selection. Specifically, the impacts of selection increase with the duration of neighborhood measurement.

**Table 4:** The “effect” of neighborhood on education level: Estimates of the percentage of the variation in adult educational attainment attributable to county of residence.

	30 or older	32 or older	34 or older	36 or older
*N*	8,626	7,752	7,244	6,676
Overall	0.094*** (0.064–0.123)	0.094*** (0.059–0.130)	0.103*** (0.064–0.141)	0.100*** (0.062–0.137)
*N*	7,005	6,649	6,305	5,889
County > 2	0.105*** (0.072–0.138)	0.103*** (0.070–0.136)	0.111*** (0.078–0.144)	0.107*** (0.069–0.146)
*N*	6,108	5,857	5,598	5,292
County > 4	0.107*** (0.071–0.143)	0.106*** (0.068–0.145)	0.117*** (0.074–0.159)	0.110*** (0.071–0.149)
*N*	5,298	5,089	4,894	4,658
County > 6	0.110*** (0.071–0.149)	0.109*** (0.071–0.147)	0.119*** (0.078–0.160)	0.117*** (0.078–0.156)
*N*	4,675	4,480	4,311	4,127
County > 8	0.111*** (0.071–0.151)	0.111*** (0.073–0.149)	0.121*** (0.078–0.164)	0.120*** (0.077–0.162)
*N*	4,136	3,967	3,813	3,648
County > 10	0.114*** (0.075–0.153)	0.116*** (0.073–0.158)	0.127*** (0.089–0.165)	0.127*** (0.081–0.173)

Note:

***<0.01

Controls: Age, gender, race, survey year. *Columns* restrict the ages of the sample; *Rows* restrict the sample to respondents who have lived in the same county for a given number of years. This table serves as a “negative test” in that current county of residence cannot causally effect completed years of schooling for adults and is meant to capture the degree to which selection effects are operating in the analysis.

## Discussion

This paper provides additional insights into the interpretation of “place effects” on health and related processes. An important question in the literature is how the timing of place effects is tied to health, educational, and socioeconomic outcomes. For example, Hicks et al. [11] provide evidence that the recency of exposure to neighborhood disadvantage has larger impacts on test scores than effects of average exposure. Because in many analyses, the measurement of place and the outcome is contemporaneous (cross-sectional data), it is often difficult to separate cumulative and short-term effects. Longitudinal data on place suffers from a different issue of dynamic selection effects. It is possible that conditioning on the length of time spent in a place in order to capture a richer place effect builds in larger selection effects.

The current paper explores these issues with longitudinal data on place from the PSID tied to measures of adult health. Our findings present a complicated picture with respect to interpreting longitudinal effects of place. We find evidence that using cross-sectional measures of place likely understates its importance in determining variation in adult health (though the effect is driven by both causal and selection effects). This suggests a need to include longitudinal measures of place in future analyses. However, we also find that using longitudinal measures of place also increases the fraction of the “effects” on health through inducing larger and dynamic selection processes into the analysis. We currently have few empirical tools to disentangle these processes. Some recent work has deployed inverse probability weighting schemes to decompose the effects of a specific place-based measure on educational attainment [12]; however, there are not yet methods to analyze and decompose the “overall” effects of place into selection and causal effects. Our paper suggests this decomposition is an essential next step to understanding the impacts of place on health and related outcomes.
